# Pest and Host Associations That Transcend Time: Assessing the Impact of Climate Change on Grape Berry Moth (*Paralobesia viteana*) and Its Hosts *Vitis riparia* and *Vitis labrusca* in North America

**DOI:** 10.1002/ece3.72612

**Published:** 2025-12-09

**Authors:** Jesús H. Gómez‐Llano, Dori Edson Nava, Fabio Castro Llanos, Flor E. Acevedo

**Affiliations:** ^1^ Universidade Federal de Pelotas (UFPEL) Pelotas Rio Grande do Sul Brazil; ^2^ Embrapa Clima Temperado Pelotas Rio Grande do Sul Brazil; ^3^ International Center for Tropical Agriculture (CIAT) Cali Valle del Cauca Colombia; ^4^ Department of Entomology The Pennsylvania State University University Park Pennsylvania USA

**Keywords:** Canada, GBM, SDM, Tortricidae, United States, viticulture

## Abstract

The grape berry moth (GBM) *Paralobesia viteana* (Clemens, 1860) (Lepidoptera: Tortricidae) is an important pest of grapes in Eastern North America. The insect is native to this region and co‐evolved with wild grapevine hosts long before the beginning of viticulture. The geographic distribution of this pest is influenced by the distribution of its hosts and by unknown environmental factors. In agriculture, species distribution models (SDMs) can help predict the effects of environmental variables and changing climate on the geographic suitability of pests and their hosts, guiding preparation for potential pest expansions. This study predicted the potential geographic distribution of GBM and two of its host plants, *Vitis labrusca* and *Vitis riparia* , across the United States (U.S.) and Canada in the current time and under two climate change scenarios (SSP2–4.5 and SSP5–8.5) and periods (2021–2040 and 2041–2060) using the Random Forest algorithm. The results show that habitat suitability for the three species was primarily determined by temperature and precipitation. The temperature annual range and the precipitation of the driest month were the variables with the greatest influence on GBM distribution, whereas the mean temperature of the warmest quarter contributed the most to *V. labrusca* and *V. riparia* SDMs*.* Shared suitable areas for GBM and its two hosts in current time predictions were 9.7% and 1.76% in the U.S. and Canada territories, respectively. In future climatic scenarios, these shared suitable areas are predicted to increase by 3.3%–4.5% in the Northeast and Midwest U.S. and by 7.8%–13% in Eastern Canada. These findings predict an increase in pest pressure in the U.S. and Canada in future climatic conditions, providing the basis for proactive pest monitoring, breeding for drought and cold grapevine tolerance, and adaptive vineyard management to mitigate the risks associated with climate change.

## Graphical abstract

This study predicted the potential geographic distribution of GBM and two of its host plants, *Vitis labrusca* and *Vitis riparia* , across the United States (U.S.) and Canada in the current time and under two climate change scenarios (SSP2–4.5 and SSP5–8.5) and periods (2021–2040 and 2041–2060) using the Random Forest algorithm. Shared suitable areas for GBM and its two hosts in current time predictions were 9.7% and 1.76% in the U.S. and Canada territories, respectively. In future climatic scenarios, these shared suitable areas are predicted to increase by 3.3%–4.5% in the Northeast and Midwest U.S. and by 7.8%–13% in Eastern Canada. These findings suggest an increase in pest pressure in the U.S. and Canada in future climatic conditions.

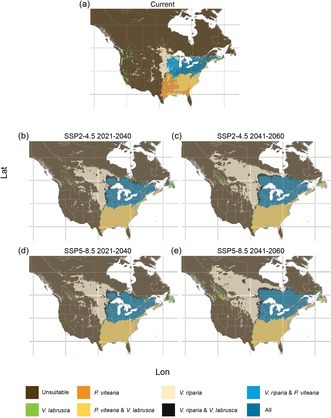

## 1 Introduction

Grape production plays a vital role in the agricultural economies of the United States (U.S.) and Canada. In the U.S., grapes are one of the highest‐value fruit crops grown across the country, with major production concentrated in California, Washington, and New York (USDA and NASS 2020). In Canada, grapes are cultivated in the Niagara Peninsula in Ontario, British Columbia, southern Quebec, and Nova Scotia (CCOVI 2020). Grape production in both countries generates employment and supports secondary industries, such as wineries and tourism, contributing significantly to the rural and local economies. The grape‐producing regions within the U.S. and Canada have contrasting environmental conditions that influence vine cultivar adaptability, disease, and pest pressure. This highlights the need to better understand the effect of environmental variables on species distributions for the design of pest management programs.

The grape berry moth (GBM) *Paralobesia viteana* (Clemens, 1860) (Lepidoptera: Tortricidae), previously known as *Endopiza viteana* Clemens, is native to Eastern North America and specializes in *Vitis* spp. *P. viteana* is considered one of the most important pests of grapes in the U.S. and Canada (Isaacs et al. 2012). The GBM larvae cause direct damage by feeding on grape clusters and indirect damage by enhancing grape vulnerability to pathogen infection, ultimately reducing crop yields and compromising the quality of juice and wine. *P. viteana* feeds on wild and cultivated grapes growing in Ontario and Quebec (Canada) and across the Eastern and parts of the Central U.S. regions, but it is absent on the west coast of both countries despite the wide availability of susceptible hosts. The geographic distribution of this insect is likely associated with environmental factors that are yet to be elucidated.

*Paralobesia viteana* evolved with native wild grape hosts that play a key role in its life cycle. This insect overwinters in the pupal stage, and the first generation of adults emerges in spring. Because some wild grape species bloom earlier than the cultivated ones, a large portion of the first‐generation adults infest wild grapes, and the subsequent generations migrate to cultivated vineyards (Taschenberg et al. 1974). In the Northeast and Midwest U.S., wild grapes commonly grow in habitats adjacent to vineyards, increasing the risk of grape berry moth infestation (Botero‐Garcés and Isaacs 2004). Experimentally, it has been demonstrated that wild grapes can reach GBM infestation levels of up to 84.9% in Michigan and between 50% and 80% in New York State (Dennehy et al. 1990; Botero‐Garcés and Isaacs 2004). More than 30 species of wild grapes have been reported in North America, inhabiting various environments, from disturbed areas to the edges of mature forests (Morano and Walker 1995; Morin et al. 2015). Some of the most common wild grape species in North America associated with GBM are the riverbank grape, *Vitis riparia* Michaux, and the fox grape, *Vitis labrusca* L. Both species are also extensively used in the development of cold‐hardy cultivars (Clark 2019; Londo and Kovaleski 2019; Köse et al. 2024).

Female GBM moths lay their eggs on flower clusters and grapes at any stage of maturity, and the emerging larvae subsequently feed on them (Clark and Dennehy 1988; Isaacs et al. 2012). When oviposition takes place on larger grapes (pea‐size or bigger), the emerged GBM larvae bore into the berries to feed and develop internally (Luciani 1987). This feeding behavior protects the larvae from some natural enemies and chemical interventions. After fully grown, the last instar larvae exit the grapes and pupate in silk chambers built in rolled grapevine leaves. The GBM life cycle takes on average 32 days at 25°C with some variation associated with the grape cultivar, and there can be 3–4 generations per season depending on temperature accumulation (Biever and Hostetter 1989; Hoffman et al. 1992; Laiton‐Jimenez et al. 2024).

Insects rely on ambient heat to regulate their body processes, making temperature a critical factor influencing their development, survival, and reproduction (Ziter et al. 2012; Sampaio et al. 2021; Boulanger et al. 2025). Humidity levels are also critical to prevent desiccation of certain living stages such as eggs and larvae. Therefore, the rise in temperatures and variations in rainfall associated with climate change can have profound effects on insect distribution and pest outbreaks (Dukes et al. 2009). For instance, warmer winters can lead to higher survival rates of overwintering insect stages, altering their phenology and accelerating their life cycles (Deutsch et al. 2018; Marshall et al. 2020; Ma et al. 2021; Lawton et al. 2022). Climate change could induce shifts, reductions, or geographic expansion of insects (Ma et al. 2021; Boulanger et al. 2025). Furthermore, changes in climate patterns could also affect the availability, quality, or susceptibility of host plants, creating complex challenges for insect conservation and for managing herbivore pests (Vagelas et al. 2024; Singh et al. 2025; Chang et al. 2025). However, the species' responses to climate change are variable and depend on a combination of abiotic and biotic factors (Robinet and Roques 2010). To predict the effects of climate change on species distribution, scientists have utilized species distribution models (SDMs) (Gillson et al. 2013; Srivastava et al. 2019; Machado Teixeira et al. 2022; Yang et al. 2025; Adhikari et al. 2025). These models use species occurrences and environmental data to predict suitable occupancy areas in current and future climatic conditions (Srivastava et al. 2019; Yao et al. 2025). Therefore, SDMs are valuable tools for forecasting species range shifts, expansions, or contractions that could be crucial in conservation and management programs (Newbold 2010; Franklin 2013; Srivastava et al. 2019). In agriculture, SDMs can help predict the effects of climate change on pests and their hosts, providing valuable insights to prepare for potential pest expansions (Kroschel et al. 2013; Wang et al. 2025; Galvão‐Silva et al. 2025).

Recent ecological niche models suggest that the influence of climate change can increase suitable areas for *V. riparia* and *V. labrusca* in the U.S. and Canada (Zhang et al. 2025). Therefore, these species can be valuable for developing cultivars better adapted to future climate change conditions. For GBM, previous studies predicted an increase in the number of generations per year under elevated temperatures and higher greenhouse gas emissions (Chen et al. 2011). Also, an increase in temperature may shift the ovipositional period due to earlier diapause termination (Tobin et al. 2008). However, there is limited information about the effect of climate change on GBM distribution across North America. Species distribution models could help predict the expansion of this significant pest in association with two of its primary grapevine hosts. Such models would be valuable for assessing potential risks to viticultural regions and informing proactive management strategies.

This study predicts the potential geographic distribution of the grape berry moth *P. viteana* and two of its host plant species, *V. labrusca* and *V. riparia* , across the U.S. and Canada in the current time and under two different climate change scenarios and periods. We hypothesize that the historical ecological association between *P. viteana* and these wild grape species will result in spatially overlapping suitable habitats under current and future climate conditions. Modeling these distributions would help identify regions of species co‐occurrence that may increase pest pressure in vineyards. It would also help determine the resilience to climate change of the species under study.

## 2 Materials and Methods

This study models the potential distribution of a grape vineyard pest, *P. viteana*, and two of its host plants, *V. riparia* and *V. labrusca* , in the U.S. and Canada. The purpose of these predictions was to identify areas potentially suitable for the establishment of each species, as well as overlapping areas under current and future climate change scenarios.

### 2.1 Species Occurrence Records

We obtained 2050 geographical coordinates for *P. viteana*, 4145 for *V. labrusca* and its variants (*Vitis latifolia* Raf., *Vitis* × *alexanderi*, *Vitis* × *novae‐angliae* , *Vitis* × *labruscana* L.H. Bailey), and 9723 for *V. riparia* from the U.S. and Canada from the following databases: The Global Biodiversity Information Facility (GBIF; https://www.gbif.org), iNaturalist (www.iNaturalist.com.mx; https://www.inaturalist.org/), and The Integrated Digitized “Biocollections” (“iDigBio”). The occurrence records were extracted using the “occ” function from the “spocc” RStudio package (Owens et al. 2023). Subsequently, the occurrences dataset was filtered to remove common spatial and temporal errors using the function “clean_coordinates” from the package “CoordinateCleaner.” (Zizka et al. 2019). With this procedure, we excluded records assigned to a province centroid, the ocean, lakes, urban areas of biodiversity institutions, zero coordinates, identical latitude/longitude, invalid coordinates, outlier coordinates, and duplicate coordinates. The filtered coordinates dataset containing 446 records for *P. viteana*, 2471 for *V. labrusca* , and 3682 for *V. riparia* was saved in a “. cvs” file for further use. To assess potential spatial autocorrelation, we visually inspected the spatial distribution of cleaned occurrence points for each species. As no strong clustering patterns were detected, no additional thinning procedures were applied.

The construction of SDMs with presence‐only data requires a characterization of the environmental background of the study area to identify locations where the species under consideration have not been recorded; the so‐called “pseudo‐absences” are used by the model to discriminate between suitable and unsuitable habitats (Renner et al. 2015). Pseudo‐absence locations were generated within the accessible area for the species, defined as the geographic region where the species has been historically documented and could feasibly disperse over evolutionary and recent ecological time (Barve et al. 2011). For the three species, we considered the entire United States and Canada as the accessible region, given their known biogeographic origin and dispersal history (Isaacs et al. 2012; Morin et al. 2015). Within this region, pseudo‐absence points were randomly drawn at a 2:1 ratio relative to presence using the “randomPoints” function in the “dismo” R package (Hijmans et al. 2023).

### 2.2 Environmental Variables

We selected 19 bioclimatic variables (BIO1–BIO19; Table 1) to predict suitable areas for the species under study using SDMs. These variables provide information on temperature and precipitation in the study area, describing annual and seasonal trends as well as extreme conditions (Nix 1986; Fick and Hijmans 2017). For current time predictions, we pre‐selected 19 bioclimatic variables generated from historical records between the years 1970 and 2000. These variables were obtained from the WorldClim database (Global Climate Data; available at WorldClim: https://www.worldclim.org/data/index.html) in a “. raster” format at a spatial resolution of 2.5 arc‐minutes (approximately 4.5 km). To reduce collinearity among the environmental variables, we fitted the model using the 19 bioclimatic variables as predictors for the occurrences of each species (*P. viteana*, *V. labrusca* , and *V. riparia* ). Subsequently, we extracted the values of each bioclimatic variable for each occurrence using the function “extract” from the “terra” R package (Hijmans et al. 2024). The Variance Inflation Factor (VIF) values for each variable were calculated using the “vifstep” function from the “usdm” R package (Naimi et al. 2014). Variables with VIF values exceeding 10 were excluded, and eight non‐collinear bioclimatic variables were retained for each species. The selected variables for *P. viteana*, *V. labrusca* , and *V. riparia* are listed separately in Table 2.

**TABLE 1:** Bioclimatic variables for the species distribution models obtained from the WorldClim database (Global Climate Data; available at WorldClim: https://www.worldclim.org/data/index.html) (Nix 1986; Fick and Hijmans 2017).

Code	Variable
BIO1	Annual mean temperature
BIO2	Mean diurnal range (mean of monthly (max temp–min temp))
BIO3	Isothermality (BIO2/BIO7) (×100)
BIO4	Temperature seasonality (standard deviation ×100)
BIO5	Max temperature of warmest month
BIO6	Min temperature of coldest month
BIO7	Temperature annual range (BIO5–BIO6)
BIO8	Mean temperature of wettest quarter
BIO9	Mean temperature of driest quarter
BIO10	Mean temperature of warmest quarter
BIO11	Mean temperature of coldest quarter
BIO12	Annual precipitation
BIO13	Precipitation of wettest month
BIO14	Precipitation of driest month
BIO15	Precipitation seasonality (coefficient of variation)
BIO16	Precipitation of wettest quarter
BIO17	Precipitation of driest quarter
BIO18	Precipitation of warmest quarter
BIO19	Precipitation of coldest quarter

**TABLE 2:** Relative contribution of the selected bioclimatic variables to the Random Forest SDMs for *Paralobesia viteana*, *Vitis labrusca* , and *Vitis riparia* .

Species	Codes	Bioclimatic variable	%IncMse	Contribution (%)
*Paralobesia viteana*	BIO7	Temperature annual range (BIO5–BIO6)	0.140	19.97
	BIO14	Precipitation of driest month	0.135	19.18
	BIO10	Mean temperature of warmest quarter	0.096	13.93
	BIO16	Precipitation of wettest quarter	0.060	8.51
	BIO9	Mean temperature of driest quarter	0.046	6.49
	BIO8	Mean temperature of wettest quarter	0.042	5.96
	BIO18	Precipitation of warmest quarter	0.037	5.27
	BIO2	Mean diurnal range (mean of monthly (max temp–min temp))	0.035	4.98
*Vitis labrusca*	BIO10	Mean temperature of warmest quarter	0.149	19.08
	BIO15	Precipitation seasonality (coefficient of variation)	0.142	16.51
	BIO9	Mean temperature of driest quarter	0.129	12.20
	BIO2	Mean diurnal range (mean of monthly (max temp–min temp))	0.079	12.11
	BIO3	Isothermality (BIO2/BIO7) (×100)	0.079	11.34
	BIO8	Mean temperature of wettest quarter	0.041	11.03
	BIO19	Precipitation of coldest quarter	0.040	9.62
	BIO18	Precipitation of warmest quarter	0.024	8.13
*Vitis riparia*	BIO10	Mean temperature of warmest quarter	0.160	22.80
	BIO9	Mean temperature of driest quarter	0.155	22.09
	BIO3	Isothermality (BIO2/BIO7) (×100)	0.126	17.98
	BIO6	Min temperature of coldest month	0.103	14.61
	BIO8	Mean temperature of wettest quarter	0.046	6.51
	BIO18	Precipitation of warmest quarter	0.042	5.91
	BIO14	Precipitation of driest month	0.036	5.12
	BIO13	Precipitation of wettest month	0.035	4.98

*Note:* Column four represents the increase in mean squared error “%IncMse” used to quantify the importance of each predictor variable in the SDMs. Column five represents the contribution of each variable through analysis of multicollinearity and AUC metrics.

The bioclimatic variables for future predictions were derived from 25 Global Climate Models from the “Coupled Model Intercomparison Project Phase 6” (CMIP6; available at WorldClim: https://www.worldclim.org/data/cmip6/cmip6climate.html) (Table S1) (O'Neill et al. 2016). These models provide climate projections based on CO2 emissions and mitigation effort scenarios up to the year 2100, known as “Shared Socioeconomic Pathways” (SSPs). For this research, we selected two SSPs (SSP2–4.5 and SSP5–8.5) for two future time intervals (2021–2040 and 2041–2060). The SSP2–4.5 is known as a stabilization scenario with the implementation of climatic policies to reduce greenhouse gas emissions (Thomson et al. 2011). In contrast, the SSP5–8.5 is a high greenhouse gas emissions pathway considered a “baseline” scenario with no specific climatic mitigation targets (Riahi et al. 2011).

### 2.3 Modeling and Projections

We used Random Forest, a powerful machine‐learning algorithm for building SDMs, especially for datasets with a limited number of occurrences (Li et al. 2015; Mi et al. 2017; Luan et al. 2020). This method creates an ensemble of hundreds of decision trees using random subset samples from the occurrences/pseudo‐absence datasets and the predictors “bioclimatic variables.” Usually, several parameters control the structure of Random Forest models, involving the configuration of each tree and the “forest,” as well as its randomness. Key parameters include the number of candidate variables randomly drawn at each split (mtry), the node size, and the number of trees in the “forest” (ntree). The optimal selection of these parameters is essential for improving model prediction accuracy and is typically achieved through tuning strategies designed to identify the best parameter values (Probst, Boulesteix, and Bischl 2019; Probst, Wright, and Boulesteix 2019). In this study, the selection of parameter values was carried out using the “train” function from the “caret” package in RStudio (Kuhn 2015). We used an ntree of 2500, a mtry of 3, and a node size of 2 with 25 replications for all SDMs made for *P. viteana*, *V. labrusca* , and *V. riparia* . All models were trained using 75% of the occurrence data, with the remaining 25% reserved for testing. For the current projections, we generated an ensemble using the 25 replications for each species' SDM. For future predictions, the ensemble was generated by combining the 25 Global Climate Models in each climatic period (2021–2040 and 2041–2060) and Representative Concentration Pathway (SSP).

The SDMs output using Random Forest provides a suitability score that ranges from 0 to 1. To facilitate the interpretation of these results, we established a threshold for each species, extracting all the thresholds obtained from Receiver Operating Characteristic (ROC) curve data using the function “roc” of the “pROC” RStudio package (Robin et al. 2011). The optimal threshold was then selected as the value minimizing the Euclidean distance to the upper‐left corner of the ROC plot (sensitivity = 1, specificity = 1), which identifies the classifier closest to the theoretical optimum (Liu et al. 2005). The suitability values for each predicted scenario and period were converted into binary values using a single species‐specific threshold derived from the current model evaluation. Values exceeding the threshold were classified as suitable (1), while those below were considered unsuitable (0). The thresholds applied were 0.195 for *P. viteana*, 0.181 for *V. riparia* , and 0.130 for *V. labrusca* .

Maps were plotted for each species using the “ggplot2” RStudio package (Wickham et al. 2025), with the suitability score ranging from 0 to 1, in a color scale gradient from dark blue to red, respectively. At last, the binary data for all the species analyzed were plotted in a single map to show the overlapped suitable areas among the three species for each period and climatic change scenario. Afterward, the suitable and unsuitable areas for all species analyzed were quantified in square kilometers using the “expanse” function from the “terra” package in RStudio (Hijmans et al. 2024).

To evaluate the model performance, we carried out the ROC curve analysis, focusing on the area under the curve (AUC) and the True Skill Statistic (TSS) (Fielding and Bell 1997). Both AUC and TSS values are well‐established metrics for assessing the accuracy of SDMs. Typically, AUC values below 0.7 indicate poor performance, values between 0.7 and 0.9 suggest moderate performance, and values exceeding 0.9 denote good performance and high accuracy (Jiménez‐Valverde 2012; Shabani et al. 2018). While TSS values go from −1 to 1, showing better performance of the model when values are close to 1 (Allouche et al. 2006).

For each species, we assessed the relative importance of the selected bioclimatic variables by analyzing multicollinearity and AUC metrics. This integrated approach enabled us to evaluate both the predictive power of the models and the significance of individual variables within the SDMs. Additionally, the permutation‐based %IncMSE metric derived from the final Random Forest model was calculated. For the three main bioclimatic variables with a major contribution to the SDMs for each species, we generated partial dependence plots using the “partial” function from the “pdp” RStudio package (Greenwell 2017). This allowed us to assess the marginal effect of each bioclimatic variable on the predicted probability of species occurrence. The results were visualized with “ggplot2” and saved as high‐resolution images (Wickham et al. 2025).

## 3 Results

### 3.1 Model Evaluation and Importance of Environmental Variables

The AUC and TSS values for all our SDMs exceeded 0.80, indicating high accuracy. For *P. viteana*, the mean AUC was 0.988 ± 0.0091 (standard deviation) and the mean TSS was 0.814 ± 0.0350; for *V. labrusca* the AUC was 0.993 ± 0.0051, and TSS was 0.912 ± 0.0124, while for *V. riparia* , the AUC and TSS were 0.994 ± 0.0034 and 0.892 ± 0.0137, respectively.

Among the environmental variables analyzed, the mean temperature of the warmest quarter (BIO10) contributed the most to *V. labrusca* and *V. riparia* SDMs and was the third most important variable for *P. viteana* (Table 2). The main variables for the SDM of *P. viteana* were the temperature annual range (BIO7) with a contribution of 20%, the precipitation of the driest month (BIO14) with 19%, and the mean temperature of the warmest quarter (BIO10) with a 14% contribution (Table 3). For *V. labrusca* SDM, the variables mean temperature of the warmest quarter (BIO10), precipitation seasonality (BIO15), and mean temperature of the driest quarter (BIO9) had the highest contribution with 19%, 16%, and 12%, respectively (Table 2). For *V. riparia* SDM, the main contributing variable was the mean temperature of the warmest quarter (BIO10) with 22.8%, followed by the mean temperature of the driest quarter (BIO9) with 22%, and isothermality (BIO3) with 1% (Table 2).

**TABLE 3:** Percentage of projected suitable areas for the grape berry moth, *Paralobesia viteana*, and its hosts *Vitis riparia* and *Vitis labrusca* , in current and future time predictions (SSP2–4.5 and SSP5–8.5; periods 2021–2040 and 2041–2060) in the United States of America and Canada.

Species	Country	Current (%)	SSP2–4.5		SSP5–8.5	
			2021–2040 (%)	2041–2060 (%)	2021–2040 (%)	2041–2060 (%)
*Paralobesia viteana*	United States of America	32.76	34.70	35.01	34.78	35.19
	Canada	3.09	10.32	14.56	10.66	16.60
*Vitis labrusca*	United States of America	22.44	36.32	36.79	36.43	37.14
	Canada	2.22	13.08	17.26	13.49	19.83
*Vitis riparia*	United States of America	20.50	23.41	23.87	23.20	24.92
	Canada	3.99	21.47	31.34	22.71	35.67
*P. viteana*, *V. labrusca* , and *V. riparia*	United States of America	9.7	14.21	13.51	14.03	13.07
	Canada	1.76	9.62	13.18	9.86	14.83

**FIGURE 1:**
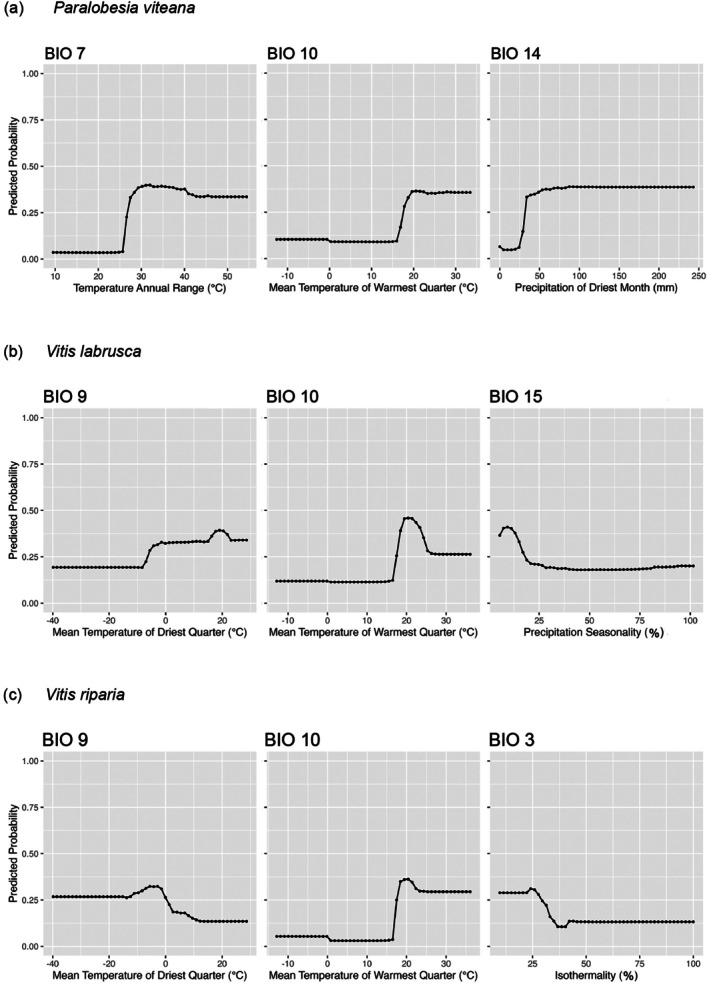
Partial dependence curves for the three main bioclimatic variables of (a) *Paralobesia viteana* SDM, (b) *Vitis labrusca* SDM, and (c). *Vitis riparia* SDM. The *y* axis represents the predicted presence probability of each species.

The partial dependence plots showed an increasing predicted probability of *P. viteana*, *V. labrusca* , and *V. riparia* presence at BIO10 values higher than 17°C and peaking at 20°C. For *P. viteana*, the predicted presence probability remained mostly stable from 20°C to 34°C; for *V. labrusca* the highest suitability ranged from 18°C to 22°C, dropped at 25°C and then remained constant until 32°C, whereas for *V. riparia*, the highest suitability ranged from 18°C to 21°C, dropped at 23°C and then remained constant until 37°C (Figure 1a–c). The predicted presence probability of *P. viteana* also increased at BIO7 (temperature annual range) values higher than 26°C, peaking at 32°C, and remained slightly lower and relatively constant until 55°C. The precipitation of the driest month (BIO14) also increased the predicted presence probability of *P. viteana* at values between 35 and 245 mm, with optimal values above 55 mm (Figure 1a). For the two *Vitis* species analyzed, the response to BIO9 (mean temperature of driest quarter) differed markedly: *V. labrusca* had increasing suitability probability at temperatures above 0°C, whereas *V. riparia* exhibited greater suitability at subzero temperatures (Figure 1b,c). Additionally, the predicted presence probability of *V. labrusca* increased with precipitation seasonality values below 13% (BIO15). *Vitis riparia* seems to adapt better to areas with low to moderate isothermality values ranging from 5% to 27% (BIO3) (Figure 1c).

### 3.2 Predicted Suitable Areas in Current and Future Conditions

**FIGURE 2:**
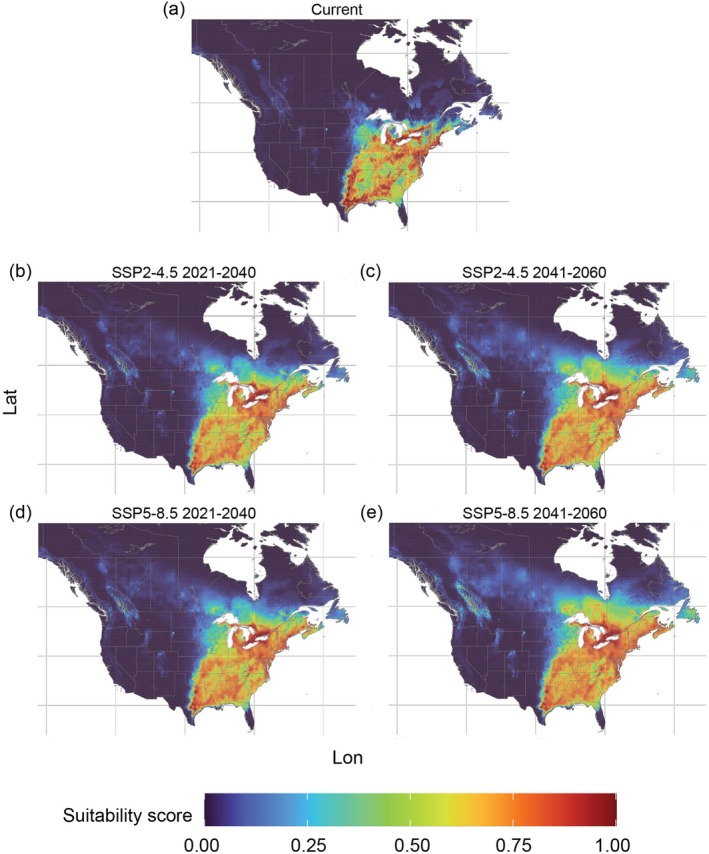
Predicted suitable areas for *Paralobesia viteana* in the (a) current period and (b–e) in future climate change scenarios SSP2–4.5 and SSP5–8.5; (b) scenario SSP2–4.5 period 2021–2060; (c) scenario SSP2–4.5 period 2041–2060; (d) scenario SSP5–8.5 period 2021–2040; (e) scenario SSP5–8.5 period 2041–2060. Panels are displayed over the North American extent (−138° to −55° W, 26° to 65° N).

The predicted suitable areas for *P. viteana* in current environmental conditions were restricted to the Eastern and parts of the Central U.S. (32.76% of territory) as well as southern Canada (Ontario and Quebec; 3.09% of territory) (Figure 2a). Future projections indicate an expansion in suitable areas, particularly under the 2041–2060 SSP5–8.5 scenario (Table 3). In the U.S., suitable areas for *P. viteana* are projected to increase by up to 2.43%, mainly in the northeastern region. In Canada, there was a predicted increase in suitable areas of 13.51%, concentrated in Ontario, Quebec, New Brunswick, Prince Edward Island, and Nova Scotia (Table 3). Additionally, future projections for *P. viteana* in the U.S. indicated minimal variation in suitable areas across both climate scenarios (SSP2–4.5 and SSP5–8.5) and periods (2021–2040 and 2041–2060) (Figure 2b–e). Suitable areas for *P. viteana* were predicted to cover 34.70% of the U.S. territory under the 2021–2040 SSP2–4.5 scenario and reach 35.19% under the 2041–2060 SSP5–8.5 scenario. In contrast, Canada exhibited differences between climate scenarios for *P. viteana*, particularly during the 2041–2060 period (Table 3). Suitable areas were predicted to cover 14.56% of the country's territory under the SSP2–4.5 scenario compared to 16.60% under SSP5–8.5 (Table 3). New suitable areas are predicted to be concentrated in Ontario, southern Quebec, New Brunswick, Prince Edward Island, and Nova Scotia, Canada, while in the U.S., the expansion of suitable areas was predicted to occur in the Northeast, particularly in the states of New York, Vermont, New Hampshire, and Maine (Figure 2b–e).

**FIGURE 3:**
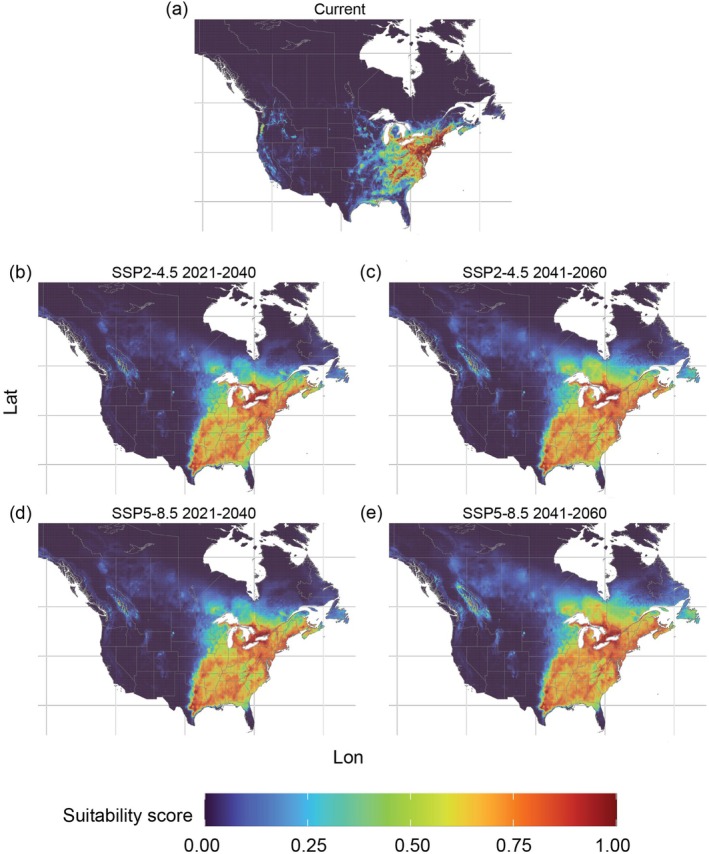
Predicted suitable areas for *Vitis labrusca* in the (a) current period and (b–e) in future climate change scenarios SSP2–4.5 and SSP5–8.5; (b) scenario SSP2–4.5 period 2021–2060; (c) scenario SSP2–4.5 period 2041–2060; (d) scenario SSP5–8.5 period 2021–2040; (e) scenario SSP5–8.5 period 2041–2060. Panels are displayed over the North American extent (−138° to −55° W, 26° to 65° N).

In the current period, the predicted suitable areas for *V. labrusca* were concentrated in the Eastern U.S., with some regions along the west coast in Washington, Oregon, California, and in southern Ontario and Quebec, and parts of Nova Scotia, Canada (Figure 3a). The future projections for this species suggest an increase in suitable areas in the U.S., up to 14.7% in the 2041–2060 SSSP5–8.5 scenario, whereas in Canada, suitable areas are projected to increase by up to 17.61% in the same scenario (Table 2). Similar results were obtained across both scenarios (SSP2–4.5 and SSP5–8.5) and periods in the predicted suitable areas for *V. labrusca* in the U.S., showing that suitable areas can cover between 36% and 37% of the territory. However, in Canada, the differences were registered mostly between the periods 2041–2060 in both scenarios, with suitable areas occupying between 17.2% (SSP2–4.5) and 19.8% (SSP5–8.5) of the country's territory. In future climate change scenarios, suitable areas for *V. labrusca* were predicted to expand to parts of the Central and Northeast U.S., as well as the Southern provinces of Quebec and Ontario, New Brunswick, Prince Edward Island, and Nova Scotia in Canada. Furthermore, the Western U.S. (Washington, Oregon, and California) presented a loss in habitat suitability for *V. labrusca* in all the projected future climate change scenarios (Figure 3b–e).

**FIGURE 4:**
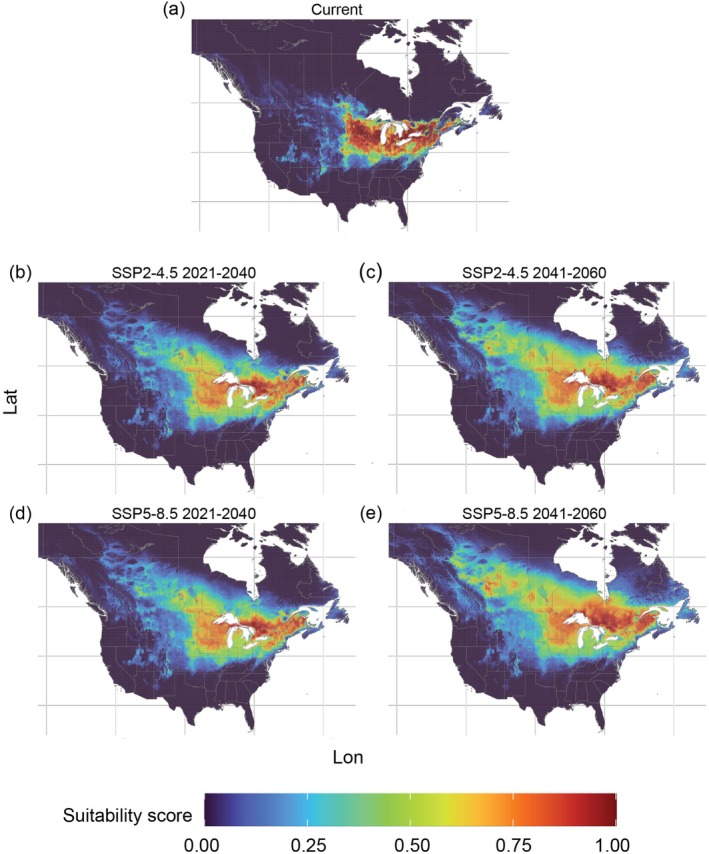
Predicted suitable areas for *Vitis riparia* in the (a) current period and (b–e) in future climate change scenarios SSP2–4.5 and SSP5–8.5; (b) scenario SSP2–4.5 period 2021–2060; (c) scenario SSP2–4.5 period 2041–2060; (d) scenario SSP5–8.5 period 2021–2040; (e) scenario SSP5–8.5 period 2041–2060. Panels are displayed over the North American extent (−138° to −55° W, 26° to 65° N).

For *V. riparia* SDM, the current time predictions showed a high suitability score (close to 1) in the Northeast and Midwest U.S. In Canada, suitable areas predicted for this species were present in the southern provinces of Ontario, Quebec, New Brunswick, and part of the Prairie provinces (Figure 4a). In the future, the suitable areas for *V. riparia* are predicted to increase in the U.S. up to 4.4% of its territory in the 2041–2060 SSP5–8.5 climate change scenario (Table 3). For this scenario, the suitable areas were predicted to increase in Canada up to 31.7% of the country's territory. Across all the future climate scenarios and periods, the predicted suitable areas in the U.S. showed similar results, ranging from 23.41% (SSP2–4.5 2021–2040) to 24.92% (SSP5–8.5 2041–2060) of the country's territory. In contrast, the predicted suitable areas in Canada showed greater variability, ranging from 21.47% (SSP2–4.5 2021–2040) to 35.67% (SSP5–8.5 2041–2060) of the country's territory. In the U.S., the expansion of suitable areas for *V. riparia* was predicted to occur in the Midwest and Northeast regions. In Canada, suitable areas were expected to expand northward, encompassing Ontario, a large portion of the Prairie Provinces, as well as Quebec and New Brunswick (Figure 4b–e).

**FIGURE 5:**
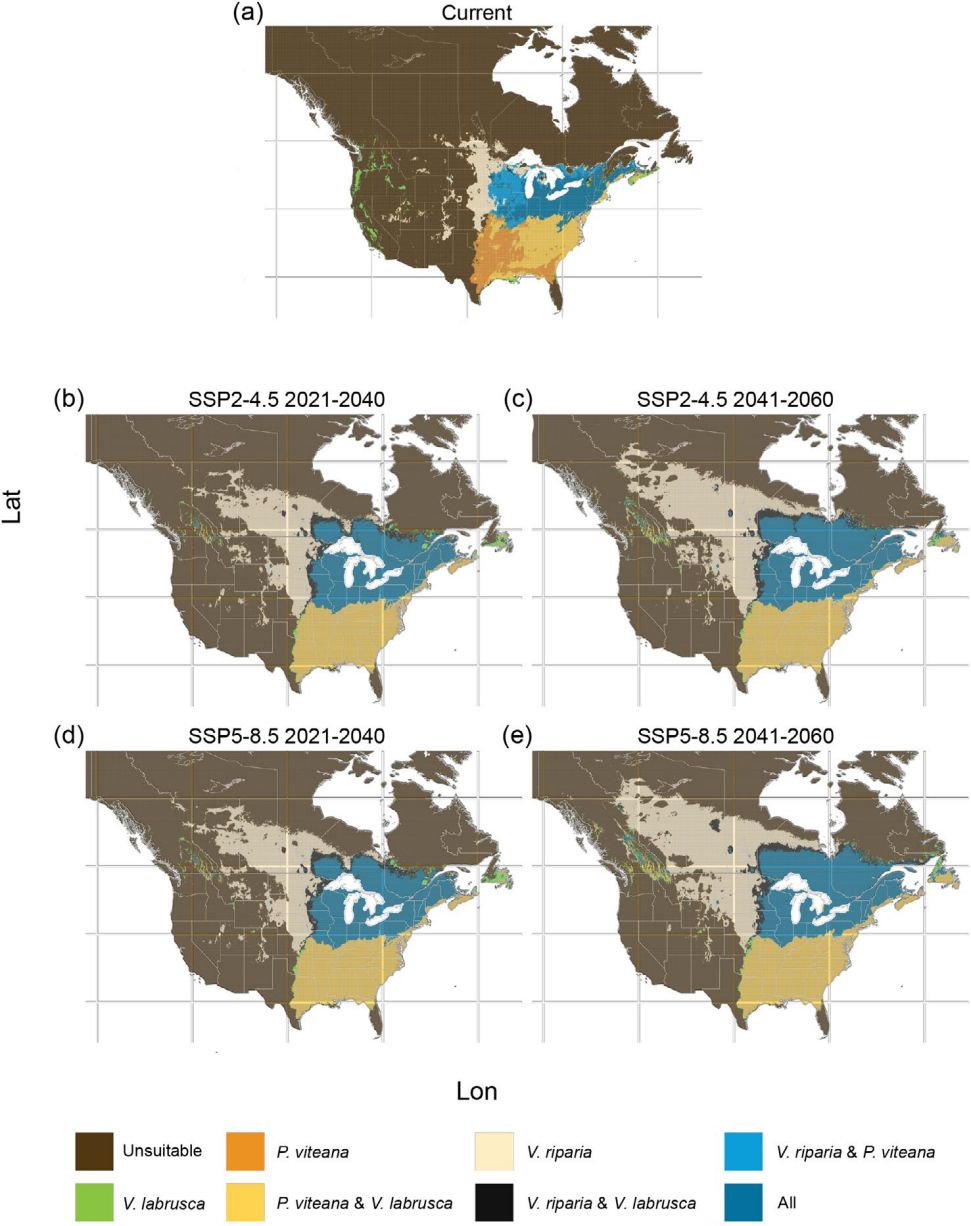
Overlapped suitable areas predicted for *Paralobesia viteana*, *Vitis labrusca* , and *Vitis riparia* , in the (a) current period and (b–e) in future climate change scenarios SSP2–4.5 and SSP5–8.5; (b) scenario SSP2–4.5 period 2021–2060; (c) scenario SSP2–4.5 period 2041–2060; (d) scenario SSP5–8.5 period 2021–2040; (e) scenario SSP5–8.5 period 2041–2060. Panels are displayed over the North American extent (−138° to −55° W, 26° to 65° N).

Our predictions suggest shared suitable areas between *P. viteana* and the grapevine hosts included in this study ( *V. labrusca* and *V. riparia* ). In the current environmental conditions, the three species were predicted to overlap in 9.7% of the U.S. territory, specifically, in the states of Wisconsin, Michigan, Illinois, Missouri, Indiana, Kentucky, Ohio, Virginia, West Virginia, Pennsylvania, New York, New Jersey, Connecticut, Massachusetts, Vermont, New Hampshire, and Maine (Figure 5a). In Canada, our current time predictions showed suitable areas for *P. viteana* and its hosts in 1.76% of the territory, especially in southern Ontario, southern Quebec, parts of New Brunswick, and Prince Edward Island (Figure 5a). Future predictions suggested an expansion of the shared suitable areas for the three species in the U.S. ranging from 13.07% (SSP5–8.5 2041–2060) to 14.21% (SSP2–4.5 2021–2040) (Table 3) of the country's territory, specifically in the states of Minnesota, Iowa, and Missouri. In Canada, the shared suitable areas are also projected to increase, ranging from 9.62% (SSP2–4.5 2021–2040) to 14.83% (SSP5–8.5 2041–2060) (Table 3), with major expansions predicted in Quebec, Ontario, and New Brunswick (Figure 5b–e). Furthermore, suitable areas for *P. viteana* and *V. labrusca* may expand to include parts of Texas, Oklahoma, Kansas, and Florida in the future under all climate scenarios (SSP2–4.5 and SSP5–8.5) and periods evaluated (2021–2040 and 2041–2060) (Figure 5b–e).

## 4 Discussion

Our distribution models yielded very high AUC values (> 0.98), which may partly reflect residual spatial autocorrelation because spatial thinning and spatially structured cross‐validation were not applied. However, the complementary TSS values (0.81–0.91) confirmed strong discriminatory performance, supporting the reliability of the suitability patterns. Thus, we interpret our results as robust indicators of relative climatic suitability, while acknowledging that AUC values may be slightly inflated.

### 4.1 The Distribution of Grape Berry Moth Is Predicted to Increase in Eastern North America Under Future Climate Change Scenarios

The grape berry moth, *P. viteana*, is distributed across Eastern North America from Ontario and New England in the north to Florida in the south, and Texas (Isely 1917; Isaacs et al. 2012). In our models, the predicted suitable areas for this pest are within temperate climate regions (Beck et al. 2023) and restricted to the Eastern and part of Central U.S. and Southern Canada. The Western U.S. is predicted to remain unsuitable for grape berry moth, despite having a temperate climate and the availability of susceptible host plants (*Vitis* spp.). The distribution of *P. viteana* was associated with bioclimatic variables of temperature and precipitation (BIO7, 14, and 10). BIO7 quantifies the amplitude of temperature fluctuation over the course of a year. Our models predict that areas with moderate to high seasonal temperature variation are suitable for *P. viteana*, with an optimal range between 30°C and 40°C (Figure 1a). Historical records of BIO7 indicate that the West Coast of the U.S. and Canada experience temperature annual ranges in the low 30s, likely influenced by the proximity to the Pacific Ocean (Zabel et al. 2014), whereas the Northeast US has values above 35°C (Table S2). Suitable areas for *P. viteana* are predicted to have optimal values of the mean temperature of the warmest quarter (BIO10) between 20°C and 33°C. This variable could vary significantly in the States that border oceans. For instance, the coastal regions of Southern California and Washington State typically have cooler annual temperatures, ranging from 10°C to 13°C, while the inland areas experience annual temperatures that range from 3°C to 46°C, and −1°C to 32°C, respectively (Zabel et al. 2014). Previous studies developed by Tobin et al. (2001) demonstrated that *P. viteana* cannot tolerate high temperatures; at 34°C, eggs, pupae, and adults begin to die under laboratory conditions. Hot summers in some regions of the West Coast may extend the thermal tolerance of this insect, making these areas unsuitable for its survival. Besides temperature, humidity seems to be an important variable for grape berry moth. Areas with precipitation lower than 25 mm in the driest month (BIO14) are predicted to be unsuitable for the establishment of this insect, whereas humid regions with precipitation higher than 35 mm in the driest month of the year seem preferable. Historical records of BIO14 in the States of Washington, Oregon, and California have means of 26, 15, and 3.8 mm, respectively, whereas those from New York, Pennsylvania, and Ohio have average values above 55.7 mm (Table S2). This information suggests that *P. viteana* may not establish in arid or semi‐arid environments with low precipitation and very high temperatures.

Under the different climate change scenarios evaluated that predict rises in temperatures, the increase in suitable habitats for *P. viteana* is projected to occur primarily in Eastern Canada. This region includes Ontario, which is the leading producer of wine and table grapes, accounting for 60%–70% of the country's wine grape production and 60% of its table grape production (Ontario.ca 2025). The potential expansion of suitable areas for *P. viteana* may represent a risk to this industry; therefore, we suggest constant monitoring of the insect in Eastern Canada.

Temperature, humidity, host availability, and photoperiod are important factors to model habitat suitability for *P. viteana*. Although there is considerable variation in diapause termination and induction in this insect, previous studies have reported that *P. viteana* requires between 205 and 215 degree days (accumulated from January first) to emerge as adults from overwintering pupa (Tobin et al. 2002). To develop from egg to adult, *P. viteana* requires an extra 424‐degree days (Tobin et al. 2001), with variations associated with grape cultivar (Laiton‐Jimenez et al. 2024), and diapause initiation was seen in 50% of the individuals at daylengths below 14 h (Nagarkatti et al. 2001). This information suggests that the number of berry moth generations per season can increase with the rise in temperatures in suitable areas (Chen et al. 2011). However, at higher latitudes, diapause initiation is expected to start earlier than in southern regions due to a faster decline in photoperiod. Another important factor for the habitat suitability of *P. viteana* is the availability and phenology of its *Vitis* hosts. The insect is commonly found feeding on flowers and developing grape clusters from wild and cultivated *Vitis* species. The degree day requirement for bloom ranges from ~300 to 350 for *V. riparia* and *V. labrusca* (Gutierrez et al. 2021), and grapes do not need to reach maturity for *P. viteana* to complete one generation. Therefore, with the predicted rise in temperatures, the insect could likely complete at least one generation per year at higher latitudes (Eastern Canada) before entering diapause.

### 4.2 Climate Change May Increase Suitable Areas for *V. labrusca* in Temperate Climate Regions and for *V. riparia* in Cold Climate Regions

*V. labrusca* is native to Eastern North America, and it is currently distributed from Ontario, Canada, to Georgia and parts of Washington State in the U.S. (Flora of North America Editorial Committee [FNA] 2016). This species hybridizes with *V. vinifera* to form the commercially important cultivars known as “Concord” and “Catawba” (Huber et al. 2016) that are grown in Eastern North America; additionally, “Concord” is grown in Washington State. Our results of *V. labrusca* distribution include the occurrences of its hybrids and therefore suggest the suitability of the commercial *Vitis* × *labruscana* L.H. Bailey cultivars.

In this study, the mean temperature of the warmest quarter (BIO10), the precipitation seasonality (BIO15), and the mean temperature of the driest quarter (BIO9) were the most important variables for predicting the distribution of *V. labrusca* (Table 2). Areas with BIO10 temperatures between 18°C and 22°C seem optimal for the establishment of this species, whereas values below 17°C seem unsuitable (Figure 1B). *V. labrusca* had a low predicted probability of occurrence in areas with extremely cold dry seasons. Areas with BIO9 temperatures above −5°C seem to improve suitability, with predicted maximum occurrence probabilities reached between 18°C and 21°C (Figure 1B). Additionally, areas with low precipitation seasonality (BIO15) seem suitable for *V. labrusca* , suggesting that this species may grow better in areas with stable rainfall patterns (Figure 1B). Own‐rooted *V. labrusca* have a relatively superficial fine root system, making them more susceptible to drought stress (Bates 2005). Moreover, Experimental drought induction using polyethylene glycol has confirmed the low drought tolerance of *V. labrusca* (Ekbic et al. 2022). In our models, the predicted suitable areas for this grapevine species overlapped with regions classified as temperate with no dry season in the Köppen–Geiger classification system (Beck et al. 2023). This is consistent with previous studies using MaxEnt models in which temperate and humid regions were found suitable for *V. labrusca* (Callen et al. 2016).

Our predictions suggest an increase in suitable areas for *V. labrusca* in the U.S. and Canada in all future climate change scenarios. The expansion is predicted to occur in the Central and Northeast U.S. and Eastern Canada. This could represent opportunities to increase the cultivated areas of *Vitis* × *labruscana* hybrids in these regions with expected economic benefits. However, our models predict a loss in habitat suitability in Washington, a primary “Concord” producing State in future climate change scenarios, which could represent substantial economic consequences. Research on drought‐resistant rootstocks may help circumvent the negative effects of climate change on grape cultivation in certain regions (Zhang et al. 2016).

The wild grape *V. riparia* is currently found in Ontario, Nova Scotia, New Brunswick, Quebec, and Manitoba in Canada (Darbyshire 2003; Catling and Mitrow 2005; Rahemi et al. 2015) as well as the Northeastern U.S., a pattern consistent with our model predictions. Similar findings were reported by Callen et al. (2016) using MaxEnt models to characterize climatic niches of *Vitis* species in North America. In our models, the mean temperature of the warmest quarter (BIO10), the mean temperature of the driest quarter (BIO9), and isothermality (BIO3) were identified as important variables for predicting the distribution of *V. riparia* . Regions where BIO10 temperatures range from 18°C to 22°C appear to provide ideal conditions for this species to establish, while temperatures below 17°C seem unfavorable (Figure 1C). *V. riparia* is predicted to have a moderate probability of occurrence in extreme cold, dry seasons with BIO9 temperatures below −15°C, but the predicted most suitable areas have BIO9 temperatures that range between −12°C and −2°C (Figure 1C). Contrarily, warm, dry seasons with a mean temperature of the driest quarter above 10°C seem unsuitable for the species. Correspondingly, the probability of *V. riparia* occurrence is projected to be higher in areas with moderate isothermality values (BIO3). These results suggest that this species may not tolerate climates with low annual temperature variability. Instead, cooler and relatively dry environments seem more suitable for the establishment of *V. riparia* .

Regions characterized by cold climates with no distinct dry season and warm summers are projected to expand under future climate change scenarios across Canada and the Midwestern U.S. (Beck et al. 2023). This correlates with the predicted increase in suitable areas for *V. riparia* in northern Canada, particularly in the Prairie provinces and the northern part of the Midwestern U.S. Indeed, predicted suitable areas for *V. riparia* , overlap with cold regions in the Köppen–Geiger classification system (Beck et al. 2023). This species is one of the most cold‐hardy grape species frequently used in grape breeding programs to enhance dormant bud cold hardiness (Londo and Kovaleski 2019) and in rootstocks of commercial cultivars. The strong cold tolerance and projected northward range expansion of *V. riparia* , suggest it could be a key resource for adapting viticulture to future climate challenges.

In summary, the projected suitable areas for *V. riparia* and *V. labrusca* are predicted to expand in future climate change scenarios, but higher latitudes in North America seem to remain unsuitable for *V. labrusca* . This coincides with previously developed ecological niche models that suggest an increase in suitable areas for *V. riparia* and *V. labrusca* in future climate conditions (Zhang et al. 2025). Both species are considered cold‐hardy and are used to improve cold hardiness in cultivated varieties (Köse et al. 2024; Atak 2025), but *V. labrusca* exhibits lower cold resistance than *V. riparia* . Previous studies using multiple physiological indices ranked *V. riparia* as the highest cold‐resistant among the American wild grape species, while *V. labrusca* was ranked in sixth position and was classified as “medium‐high” resistance (Zhang et al. 2012). This correlates with the increase in *V. labrusca* 's habitat suitability with higher BIO9 values, unlike *V. riparia* , which shows the opposite trend.

### 4.3 *P. viteana*, *V. labrusca* , and *V. riparia* Have Predicted Overlapping Suitable Areas in Current and Future Climate Conditions

The American grape berry moth is native to Eastern North America, and its distribution is highly linked to the distribution of its grapevine hosts. The insect co‐evolved with wild grape species long before grapes were commercially cultivated in the region. All grape cultivars appear to be susceptible to grape berry moth, and no natural resistance has been documented from wild grape species. Consequently, the potential distribution of this pest may be closely associated with the geographic range of its wild host plants.

The results of this study support our hypothesis of predicted overlapping suitable areas for *P. viteana* and two of its hosts, *V. labrusca* and *V. riparia* , in current and future climate conditions. In our models, the distribution of *P. viteana* closely mirrors the distribution of *V. labrusca* in Eastern North America (Figure 5). This suggests that suitable areas for the commercial cultivation of *Vitis* × *labruscana* hybrids may always be at risk of grape berry moth infestation, except for vineyards on the West Coast of the U.S. and Canada and some areas in Nova Scotia (Figure 5A). The three species are predicted to co‐occur in the Northeast and Midwest U.S. and Eastern Canada in current conditions occupying 9.7% and 1.72% of their respective national territories. These shared suitable areas are projected to expand under future climate change scenarios, reaching up to 14.83% in Eastern Canada and up to 14.21% in the Midwest U.S. The predicted expansion of suitable habitats for both wild grape species and *P. viteana* under future climate scenarios highlights the dual nature of climate change impacts: while it may create opportunities for viticulture in previously unsuitable areas, it could also increase the risk of pest proliferation.

Information on the predicted co‐occurrence areas for *P. viteana* and its host plants can be valuable for designing pest management strategies. The models can help identify regions with potential suitability for grape cultivation but not for the pest, guiding vineyard site selection. The models also predict areas at greater risk of insect infestation, which is valuable for prioritizing surveillance efforts and guiding early detection programs in viticultural areas. The predicted future suitability under different climate scenarios can also help anticipate range shifts and emerging pest pressure. When integrated with phenology and degree‐day models, these spatial predictions can improve the timing of management interventions, ultimately supporting proactive pest management strategies. Nevertheless, for practical applications, these models must be validated with field data.

### 4.4 Study Limitations

Correlative SDMs have inherent limitations that should be acknowledged (Lissovsky et al. 2021). First, the accuracy of the predictions is constrained by the quality and spatial representativeness of species occurrence data. Although we applied extensive filtering to remove erroneous, duplicate, and imprecise records, residual spatial bias in the GBIF dataset likely persists, as records are often concentrated in easily accessible areas or regions of agricultural and economic interest such as vineyards. This uneven sampling effort can skew model calibration toward well‐surveyed environments, potentially underrepresenting suitable conditions elsewhere. Second, the generation of pseudo‐absence data introduces additional uncertainty, since true absences are rarely known and pseudo‐absences may overlap with unrecorded presences or unsuitable environments, influencing the discriminatory capacity of the models. Third, the correlative nature of the modeling framework assumes that current species–environment relationships are stable across space and time. This assumption may not hold under microclimates, changing environmental conditions or in regions with novel climate combinations, leading to extrapolation uncertainty (Owens et al. 2013). This is particularly relevant for projections in northern areas, where *P. viteana* and its host species are sparsely recorded, and climatic conditions diverge from those represented in the training data. Fourth, our models only used a standard set of bioclimatic variables as predictors. Although it is well known that temperature and humidity/water availability are critical factors for the survival and growth of insects and plants, the inclusion of additional important factors could have improved the accuracy of the models. These factors include the frost‐free period length, growing degree‐day accumulation for each species at different latitudes, soil characteristics (moisture, texture, pH), land use (proportion of cultivated areas, forest cover, and urban areas), moisture index (aridity index), and topographic parameters (land elevation), among others. The combination of correlative SDMs and mechanistic models that include species‐specific thermal limits, heat indexes, the effect of photoperiod on phenology and insect diapause, and plant water balance, among others, would likely improve prediction accuracy. Consequently, the model predictions in this study help generate testable hypotheses about the effect of environmental variables on the distribution of the three species tested, guiding future mechanistic or field‐based studies; however, they should not be interpreted as definitive extrapolations of the species distributional limits.

## 5 Conclusion

Climate change may increase the suitable areas for the grape berry moth (*P. viteana*) and two grapevine host species: *V. riparia* , and *V. labrusca* , with potential significant implications for viticulture in North America. Our findings indicate that suitable habitats for both wild grape species are projected to expand, particularly in central Canada and the Midwestern U.S., driven by changes in bioclimatic variables of temperature and precipitation. This expansion could enhance grape cultivation in regions previously unsuitable for viticulture, offering economic opportunities. However, the potential increase in suitable habitats for *P. viteana* may pose a significant risk, threatening grape production. The potential for *P. viteana* to thrive under warmer conditions, coupled with the expansion of its wild grape hosts, underscores the need for proactive monitoring and the implementation of management strategies. In conclusion, the interplay between climate change, wild grape distribution, and *P. viteana* dynamics presents both opportunities and challenges for North American viticulture. Strategic planning, including pest monitoring, proactive breeding programs for drought resistance and cold hardiness, and adaptive management practices, will be essential to mitigate risks and expand viticulture in a changing climate. Future research should continue to explore the ecological and economic implications of habitat shifts to ensure the resilience and sustainability of the grape and wine industry.

## Author Contributions

**Jesús H. Gómez‐Llano:** conceptualization (lead), data curation (lead), formal analysis (lead), investigation (lead), methodology (lead), visualization (lead), writing – original draft (lead), writing – review and editing (equal). **Dori Edson Nava:** funding acquisition (equal), project administration (equal), resources (equal), supervision (equal), writing – review and editing (equal). **Fabio Castro Llanos:** methodology (equal). **Flor E. Acevedo:** conceptualization (equal), funding acquisition (equal), project administration (equal), resources (equal), supervision (equal), validation (equal), writing – review and editing (lead).

## Funding

The author J. H. Gomez received financial support from the Coordination for the Improvement of Higher Education Personnel (CAPES)—Financing Code 001 for the development of this study. The author D. E. Nava received financial support from the National Council for Scientific and Technological Development (CNPq), Grant #310233/2020‐8, for the development of this study. Flor Acevedo received funding from the Penn State Department of Entomology, the Penn State College of Agricultural Sciences startup package, and the USDA National Institute of Food and Agriculture and Hatch Appropriations under projects #PEN04757, #PEN04770, and PEN4962 for this study. The funders had no role in study design, data collection and analysis, decision to publish, or preparation of the manuscript.

## Ethics Statement

The authors have nothing to report.

## Conflicts of Interest

The authors declare no conflicts of interest.

## Supporting information


**Table S1:** ece372612‐sup‐0001‐TableS1.xlsx.

**Table S2:** ece372612‐sup‐0002‐TableS2.csv.

## Data Availability

All relevant data are within the manuscript and supplementary files. The raw data of the occurrences used in the construction of the SDMs are publicly available in the Zenodo repository at the following DOI: https://zenodo.org/records/15857108.

## References

[ece372612-bib-0001] Adhikari, S. , V. Srivastava , T. Wist , and S. D. Eigenbrode . 2025. “Charting the Course of Invasion: Ensemble Species Distribution Models Predict the Range Expansion of a Newly Invasive Aphid Pest *Metopolophium festucae cerealium* in North America.” Crop Protection 190: 107042. 10.1016/j.cropro.2024.107042.

[ece372612-bib-0002] Allouche, O. , A. Tsoar , and R. Kadmon . 2006. “Assessing the Accuracy of Species Distribution Models: Prevalence, Kappa and the True Skill Statistic (TSS).” Journal of Applied Ecology 43: 1223–1232. 10.1111/j.1365-2664.2006.01214.x.

[ece372612-bib-0003] Atak, A. 2025. “Vitis Species for Stress Tolerance/Resistance.” Genetic Resources and Crop Evolution 72: 2425–2444. 10.1007/s10722-024-02106-z.

[ece372612-bib-0004] Barve, N. , V. Barve , A. Jiménez‐Valverde , et al. 2011. “The Crucial Role of the Accessible Area in Ecological Niche Modeling and Species Distribution Modeling.” Ecological Modelling 222: 1810–1819. 10.1016/j.ecolmodel.2011.02.011.

[ece372612-bib-0005] Bates, T. 2005. “Grapevine Root Biology and Rootstock Selection in Eastern US.” In Grapevine Rootstock: Current Use, Research, and Application, 8–14. Mid‐America Viticulture and Enology Center Southwest Missouri State Uniersity.

[ece372612-bib-0006] Beck, H. E. , T. R. McVicar , N. Vergopolan , et al. 2023. “High‐Resolution (1 km) Köppen–Geiger Maps for 1901–2099 Based on Constrained CMIP6 Projections.” Scientific Data 10: 724. 10.1038/s41597-023-02549-6. 37872197 PMC10593765

[ece372612-bib-0007] Biever, K. D. , and D. L. Hostetter . 1989. “Phenology and Pheromone Trap Monitoring of the Grape Berry Moth, *Endopiza viteana* Clemens (Lepidoptera: Tortricidae) in Missouri.” Journal of Entomological Science 24: 472–481. 10.18474/0749-8004-24.4.472.

[ece372612-bib-0008] Botero‐Garcés, N. , and R. Isaacs . 2004. “Influence of Uncultivated Habitats and Native Host Plants on Cluster Infestation by Grape Berry Moth, *Endopiza viteana* Clemens (Lepidoptera: Tortricidae), in Michigan Vineyards.” Environmental Entomology 33: 310–319. 10.1603/0046-225X-33.2.310.

[ece372612-bib-0009] Boulanger, Y. , A. Desaint , V. Martel , et al. 2025. “Recent Climate Change Strongly Impacted the Population Dynamic of a North American Insect Pest Species.” PLOS Climate 4: e0000488. 10.1371/journal.pclm.0000488.

[ece372612-bib-0010] Callen, S. T. , L. L. Klein , and A. J. Miller . 2016. “Climatic Niche Characterization of 13 North American *Vitis* Species.” American Journal of Enology and Viticulture 67: 339–349. 10.5344/ajev.2016.15110.

[ece372612-bib-0011] Catling, P. M. , and G. Mitrow . 2005. “The Dune Race of *Vitis riparia* in Ontario: Taxonomy, Conservation and Biogeography.” Canadian Journal of Plant Science 85: 407–415. 10.4141/P03-084.

[ece372612-bib-0012] CCOVI . 2020. Cool Climate Oenology and Viticulture Institute 2020–21 Year in Review. Brock University.

[ece372612-bib-0013] Chang, X. , Y. Yang , L. A. Ashton , H. Pang , and S. Xing . 2025. “Understanding Climate Change Response of Plant–Insect Herbivore Interactions From Ecological Traits.” Biological Journal of the Linnean Society 144: blae130. 10.1093/biolinnean/blae130.

[ece372612-bib-0014] Chen, S. , S. J. Fleischer , and M. C. Saunders . 2011. “Projecting Insect Voltinism Under High and Low Greenhouse Gas Emission Conditions.” Environmental Entomology 40: 505–515. 10.1603/EN10099. 22251628

[ece372612-bib-0015] Clark, L. G. , and T. J. Dennehy . 1988. “Oviposition Behavior of Grape Berry Moth.” Entomologia Experimentalis et Applicata 47: 223–230. 10.1111/j.1570-7458.1988.tb01140.x.

[ece372612-bib-0016] Clark, M. D. 2019. “Development of Cold Climate Grapes in the Upper Midwestern U.S.” In Plant Breeding Reviews, 31–60. John Wiley & Sons.

[ece372612-bib-0017] Darbyshire, S. J. 2003. Inventory of Canadian Agricultural Weeds. Agriculture and Agri‐Food Canada.

[ece372612-bib-0018] Dennehy, T. J. , C. J. Hoffman , and J. P. Nyrop . 1990. “Development of Low‐Spray, Biological and Pheromone Approaches for Control of Grape Berry Moth, *Endopiza viteana* Clemens, in the Eastern United States.” In Monitoring and Integrated Management of Arthropod Pests of Small Fruit Crops, edited by N. J. Bostanian , L. T. Wilson , and T. J. Dennehy , 261–282. Intercept.

[ece372612-bib-0019] Deutsch, C. A. , J. J. Tewksbury , M. Tigchelaar , et al. 2018. “Increase in Crop Losses to Insect Pests in a Warming Climate.” Science 361: 916–919. 10.1126/science.aat3466. 30166490

[ece372612-bib-0020] Dukes, J. S. , J. Pontius , D. Orwig , et al. 2009. “Responses of Insect Pests, Pathogens, and Invasive Plant Species to Climate Change in the Forests of Northeastern North America: What Can We Predict?” Canadian Journal of Forest Research 39: 231–248. 10.1139/X08-171.

[ece372612-bib-0021] Ekbic, H. B. , İ. Gecene , and E. Ekbic . 2022. “Determination of the Tolerance of Fox Grapes (*Vitis labrusca* L.) to Drought Stress by PEG Application In Vitro.” Erwerbs‐Obstbau 64: 87–94. 10.1007/s10341-022-00669-8.

[ece372612-bib-0022] Fick, S. E. , and R. J. Hijmans . 2017. “WorldClim 2: New 1‐km Spatial Resolution Climate Surfaces for Global Land Areas.” International Journal of Climatology 37: 4302–4315. 10.1002/joc.5086.

[ece372612-bib-0023] Fielding, A. H. , and J. F. Bell . 1997. “A Review of Methods for the Assessment of Prediction Errors in Conservation Presence/Absence Models.” Environmental Conservation 24: 38–49. 10.1017/S0376892997000088.

[ece372612-bib-0024] Flora of North America Editorial Committee . 2016. Flora of North America North of Mexico: Vitaceae to Garryaceae. Oxford University Press.

[ece372612-bib-0025] Franklin, J. 2013. “Species Distribution Models in Conservation Biogeography: Developments and Challenges.” Diversity and Distributions 19: 1217–1223. 10.1111/ddi.12125.

[ece372612-bib-0026] Galvão‐Silva, F. L. , J. H. Gómez Llano , A. L. Lima , C. R. de Jesus , R. Adaime , and D. E. Nava . 2025. “Species Distribution Models Reveal Restricted Areas for Biological Control of *Bactrocera carambolae* by Its Parasitoid *Fopius arisanus* in Brazil.” Biological Control 204: 105752. 10.1016/j.biocontrol.2025.105752.

[ece372612-bib-0027] Gillson, L. , T. P. Dawson , S. Jack , and M. A. McGeoch . 2013. “Accommodating Climate Change Contingencies in Conservation Strategy.” Trends in Ecology & Evolution 28: 135–142. 10.1016/j.tree.2012.10.008. 23146578

[ece372612-bib-0028] Greenwell, B. M. 2017. “pdp: An R Package for Constructing Partial Dependence Plots.” R Journal 9: 421. 10.32614/RJ-2017-016.

[ece372612-bib-0029] Gutierrez, B. , H. Schwaninger , V. Meakem , J. Londo , and G. Y. Zhong . 2021. “Phenological Diversity in Wild and Hybrid Grapes (*Vitis*) From the USDA‐ARS Cold‐Hardy Grape Collection.” Scientific Reports 11: 24292. 10.1038/s41598-021-03783-x. 34934135 PMC8692325

[ece372612-bib-0030] Hijmans, R. J. , R. Bivand , E. Pebesma , and M. D. Sumner . 2024. “terra: Spatial Data Analysis.”

[ece372612-bib-0031] Hijmans, R. J. , S. Phillips , J. Elith , and J. Leathwick . 2023. “dismo: Species Distribution Modeling.”

[ece372612-bib-0032] Hoffman, C. J. , T. J. Dennehy , and J. P. Nyrop . 1992. “Phenology, Monitoring, and Control Decision Components of the Grape Berry Moth (*Endopiza viteana*) Risk Assessment Program in New York.” Journal of Economic Entomology 85: 2218–2227. 10.1093/jee/85.6.2218.

[ece372612-bib-0033] Huber, F. , F. Röckel , F. Schwander , et al. 2016. “A View Into American Grapevine History: *Vitis vinifera* cv. ‘Sémillon’ Is an Ancestor of ‘Catawba’ and ‘Concord’.” VITIS—Journal of Grapevine Research 55: 53–56. 10.5073/vitis.2016.55.53-56.

[ece372612-bib-0034] Isaacs, R. , L. A. F. Teixeira , P. E. Jenkins , N. B. Neerdaels , G. M. Loeb , and M. C. Saunders . 2012. “Biology and Management of Grape Berry Moth in North American Vineyard Ecosystems.” In Arthropod Management in Vineyards: Pests, Approaches, and Future Directions, edited by N. J. Bostanian , C. Vincent , and R. Isaacs , 361–381. Springer.

[ece372612-bib-0035] Isely, D. 1917. Control of the Grape‐Berry Moth in the Erie‐Chautauqua Grape Belt. U.S. Department of Agriculture.

[ece372612-bib-0036] Jiménez‐Valverde, A. 2012. “Insights Into the Area Under the Receiver Operating Characteristic Curve (AUC) as a Discrimination Measure in Species Distribution Modelling.” Global Ecology and Biogeography 21: 498–507. 10.1111/j.1466-8238.2011.00683.x.

[ece372612-bib-0037] Köse, B. , Y. Uray , K. Bayram , and F. Türk . 2024. “Cold Hardiness Degrees of Some *Vitis vinifera* L. and *Vitis labrusca* L. Cultivars Grown in Temperate Climate Condition.” Rendiconti Lincei. Scienze Fisiche e Naturali 35: 253–262. 10.1007/s12210-024-01224-1.

[ece372612-bib-0038] Kroschel, J. , M. Sporleder , H. E. Z. Tonnang , et al. 2013. “Predicting Climate‐Change‐Caused Changes in Global Temperature on Potato Tuber Moth *Phthorimaea operculella* (Zeller) Distribution and Abundance Using Phenology Modeling and GIS Mapping.” Agricultural and Forest Meteorology 170: 228–241. 10.1016/j.agrformet.2012.06.017.

[ece372612-bib-0039] Kuhn, M. 2015. “A Short Introduction to the Caret Package.” R Foundation for Statistical Computing 1: 1–10.

[ece372612-bib-0040] Laiton‐Jimenez, L. , F. Samiksha , and F. E. Acevedo . 2024. “Biology and Life Table Parameters of *Paralobesia viteana* (Lepidoptera: Tortricidae), Grown on Different Grape Cultivars.” Journal of Economic Entomology 117: 1152–1163. 10.1093/jee/toae080. 38691142

[ece372612-bib-0041] Lawton, D. , A. S. Huseth , G. G. Kennedy , et al. 2022. “Pest Population Dynamics Are Related to a Continental Overwintering Gradient.” Proceedings of the National Academy of Sciences 119: e2203230119. 10.1073/pnas.2203230119. PMC9477387 36067290

[ece372612-bib-0042] Li, Z. , Z. Ye , R. Wan , and C. Zhang . 2015. “Model Selection Between Traditional and Popular Methods for Standardizing Catch Rates of Target Species: A Case Study of Japanese Spanish Mackerel in the Gillnet Fishery.” Fisheries Research 161: 312–319. 10.1016/j.fishres.2014.08.021.

[ece372612-bib-0043] Lissovsky, A. A. , S. V. Dudov , and E. V. Obolenskaya . 2021. “Species‐Distribution Modeling: Advantages and Limitations of Its Application. 1. General Approaches.” Biology Bulletin Reviews 11: 254–264. 10.1134/S2079086421030075.

[ece372612-bib-0044] Liu, C. , P. M. Berry , T. P. Dawson , and R. G. Pearson . 2005. “Selecting Thresholds of Occurrence in the Prediction of Species Distributions.” Ecography 28: 385–393. 10.1111/j.0906-7590.2005.03957.x.

[ece372612-bib-0045] Londo, J. P. , and A. P. Kovaleski . 2019. “Deconstructing Cold Hardiness: Variation in Supercooling Ability and Chilling Requirements in the Wild Grapevine *Vitis riparia* .” Australian Journal of Grape and Wine Research 25: 276–285. 10.1111/ajgw.12389.

[ece372612-bib-0046] Luan, J. , C. Zhang , B. Xu , Y. Xue , and Y. Ren . 2020. “The Predictive Performances of Random Forest Models With Limited Sample Size and Different Species Traits.” Fisheries Research 227: 105534. 10.1016/j.fishres.2020.105534.

[ece372612-bib-0047] Luciani, M. A. 1987. “The Biology of the Grape Berry Moth, *Endopiza viteana* (Clemens) (Lepidoptera: Tortricidae) in Southern Ontario.” M.S. Thesis, University of Guelph.

[ece372612-bib-0048] Ma, C.‐S. , W. Zhang , Y. Peng , et al. 2021. “Climate Warming Promotes Pesticide Resistance Through Expanding Overwintering Range of a Global Pest.” Nature Communications 12: 5351. 10.1038/s41467-021-25505-7. PMC8429752 34504063

[ece372612-bib-0049] Machado Teixeira, C. , A. Peter Krüger , D. E. Nava , and F. R. Mello Garcia . 2022. “Global Potential Distribution of *Anastrepha grandis* (Diptera, Tephritidae) Under Climate Change Scenarios.” Crop Protection 151: 105836. 10.1016/j.cropro.2021.105836.

[ece372612-bib-0050] Marshall, K. E. , K. Gotthard , and C. M. Williams . 2020. “Evolutionary Impacts of Winter Climate Change on Insects.” Current Opinion in Insect Science 41: 54–62. 10.1016/j.cois.2020.06.003. 32711362

[ece372612-bib-0051] Mi, C. , F. Huettmann , Y. Guo , X. Han , and L. Wen . 2017. “Why Choose Random Forest to Predict Rare Species Distribution With Few Samples in Large Undersampled Areas? Three Asian Crane Species Models Provide Supporting Evidence.” PeerJ 5: e2849. 10.7717/peerj.2849. 28097060 PMC5237372

[ece372612-bib-0052] Morano, L. D. , and M. A. Walker . 1995. “Soils and Plant Communities Associated With Three *Vitis* Species.” American Midland Naturalist 134: 254–263. 10.2307/2426296.

[ece372612-bib-0053] Morin, N. R. , L. Brouillet , and G. A. Levin . 2015. “Flora of North America North of Mexico.” Rodriguésia 66: 973–981. 10.1590/2175-7860201566416.

[ece372612-bib-0054] Nagarkatti, S. , P. C. Tobin , and M. C. Saunders . 2001. “Diapause Induction in the Grape Berry Moth (Lepidoptera: Tortricidae).” Environmental Entomology 30: 540–544. 10.1603/0046-225X-30.3.540. 22182539

[ece372612-bib-0055] Naimi, B. , N. A. S. Hamm , T. A. Groen , A. K. Skidmore , and A. G. Toxopeus . 2014. “Where Is Positional Uncertainty a Problem for Species Distribution Modelling?” Ecography 37: 191–203. 10.1111/j.1600-0587.2013.00205.x.

[ece372612-bib-0056] Newbold, T. 2010. “Applications and Limitations of Museum Data for Conservation and Ecology, With Particular Attention to Species Distribution Models.” Progress in Physical Geography 34: 3–22. 10.1177/0309133309355630.

[ece372612-bib-0057] Nix, H. A. 1986. “A Biogeographic Analysis of Australian Elapid Snakes.” Atlas of Elapid Snakes of Australia 7: 4–15.

[ece372612-bib-0058] O'Neill, B. C. , C. Tebaldi , D. P. Van Vuuren , et al. 2016. “The Scenario Model Intercomparison Project (ScenarioMIP) for CMIP6.” Geoscientific Model Development 9: 3461–3482.

[ece372612-bib-0059] Ontario.ca . 2025. “Grapes.” Government of Ontario. Accessed March 19, 2025. http://www.ontario.ca/page/grapes.

[ece372612-bib-0060] Owens, H. , V. Barve , and S. Chamberlain . 2023. “spocc: Interface to Species Occurrence Data Sources.”

[ece372612-bib-0061] Owens, H. L. , L. P. Campbell , L. L. Dornak , et al. 2013. “Constraints on Interpretation of Ecological Niche Models by Limited Environmental Ranges on Calibration Areas.” Ecological Modelling 263: 10–18. 10.1603/0046-225X-30.3.540.

[ece372612-bib-0062] Probst, P. , A.‐L. Boulesteix , and B. Bischl . 2019. “Tunability: Importance of Hyperparameters of Machine Learning Algorithms.” Journal of Machine Learning Research 20: 1–32.

[ece372612-bib-0063] Probst, P. , M. N. Wright , and A.‐L. Boulesteix . 2019. “Hyperparameters and Tuning Strategies for Random Forest.” Wiley Interdisciplinary Reviews: Data Mining and Knowledge Discovery 9: e1301. 10.1002/widm.1301.

[ece372612-bib-0064] Rahemi, A. , A. Dale , H. Fisher , et al. 2015. “Distribution of Pests on *Vitis riparia* in Sandy Soils of the South‐Western Ontario.” Journal of Plant Studies 4: 21.

[ece372612-bib-0065] Renner, I. W. , J. Elith , A. Baddeley , et al. 2015. “Point Process Models for Presence‐Only Analysis.” Methods in Ecology and Evolution 6: 366–379. 10.1111/2041-210X.12352.

[ece372612-bib-0066] Riahi, K. , S. Rao , V. Krey , et al. 2011. “RCP 8.5—A Scenario of Comparatively High Greenhouse Gas Emissions.” Climatic Change 109: 33–57. 10.1007/s10584-011-0149-y.

[ece372612-bib-0067] Robin, X. , N. Turck , A. Hainard , et al. 2011. “pROC: An Open‐Source Package for R and S+ to Analyze and Compare ROC Curves.” BMC Bioinformatics 12: 77. 10.1186/1471-2105-12-77. 21414208 PMC3068975

[ece372612-bib-0068] Robinet, C. , and A. Roques . 2010. “Direct Impacts of Recent Climate Warming on Insect Populations.” Integrative Zoology 5: 132–142. 10.1111/j.1749-4877.2010.00196.x. 21392331

[ece372612-bib-0069] Sampaio, F. , F. S. Krechemer , and C. A. Marchioro . 2021. “The Hotter the Better? Climate Change and Voltinism of *Spodoptera eridania* Estimated With Different Methods.” Journal of Thermal Biology 98: 102946. 10.1016/j.jtherbio.2021.102946. 34016363

[ece372612-bib-0070] Shabani, F. , L. Kumar , and M. Ahmadi . 2018. “Assessing Accuracy Methods of Species Distribution Models: AUC, Specificity, Sensitivity and the True Skill Statistic.” Global Journal of Human‐Social Science: B Geography, Geo‐Sciences, Environmental Science & Disaster Management 18: 1–13.

[ece372612-bib-0071] Singh, A. , S. Pandey , and A. Kumar . 2025. Climate Change and Biotic Factors: A Molecular Approach. CRC Press.

[ece372612-bib-0072] Srivastava, V. , V. Lafond , and V. C. Griess . 2019. “Species Distribution Models (SDM): Applications, Benefits and Challenges in Invasive Species Management.” CABI Reviews 2019: 1–13. 10.1079/PAVSNNR201914020.

[ece372612-bib-0073] Taschenberg, E. F. , R. T. Cardé , and W. L. Roelofs . 1974. “Sex Pheromone Mass Trapping and Mating Disruption for Control of Redbanded Leafroller and Grape Berry Moths in Vineyards.” Environmental Entomology 3: 239–242. 10.1093/ee/3.2.239.

[ece372612-bib-0074] Thomson, A. M. , K. V. Calvin , S. J. Smith , et al. 2011. “RCP4.5: A Pathway for Stabilization of Radiative Forcing by 2100.” Climatic Change 109: 77–94. 10.1007/s10584-011-0151-4.

[ece372612-bib-0075] Tobin, P. C. , S. Nagarkatti , G. Loeb , and M. C. Saunders . 2008. “Historical and Projected Interactions Between Climate Change and Insect Voltinism in a Multivoltine Species.” Global Change Biology 14: 951–957. 10.1111/j.1365-2486.2008.01561.x.

[ece372612-bib-0076] Tobin, P. C. , S. Nagarkatti , and M. C. Saunders . 2001. “Modeling Development in Grape Berry Moth (Lepidoptera: Tortricidae).” Environmental Entomology 30: 692–699. 10.1603/0046-225X-30.4.692.

[ece372612-bib-0077] Tobin, P. C. , S. Nagarkatti , and M. C. Saunders . 2002. “Diapause Maintenance and Termination in Grape Berry Moth (Lepidoptera: Tortricidae).” Environmental Entomology 31: 708–713. 10.1603/0046-225X-31.4.708.

[ece372612-bib-0078] USDA , and NASS . 2020. Noncitrus Fruits and Nuts 2019 Summary. United States Department of Agriculture, National Agricultural Statistics Service.

[ece372612-bib-0079] Vagelas, I. , G. Michail , and P. Madesis . 2024. “Pests and Diseases: A Global Threat to Plants.” In Microbial Biostimulants. Apple Academic Press.

[ece372612-bib-0080] Wang, R. , N. Wu , Z. Shi , et al. 2025. “BIOMOD2 for Evaluating the Changes in the Spatiotemporal Distribution of *Locusta migratoria tibetensis* Chen in the Qinghai‐Tibet Plateau Under Climate Change.” Global Ecology and Conservation 58: e03508. 10.1016/j.gecco.2025.e03508.

[ece372612-bib-0081] Wickham, H. , W. Chang , and L. Henry . 2025. “ggplot2: Create Elegant Data Visualisations Using the Grammar of Graphics.”

[ece372612-bib-0082] Yang, M. , J. Yu , Y. Wang , et al. 2025. “Potential Global Distributions of an Important Aphid Pest, *Rhopalosiphum padi* : Insights From Ensemble Models With Multiple Variables.” Journal of Economic Entomology 118: 576–588. 10.1093/jee/toae237. 39800802

[ece372612-bib-0083] Yao, W. , J. Yang , Y. Ma , L. Liu , E. Shang , and S. Zhang . 2025. “Habitat Suitability Assessment of Key Wildlife in Hainan Tropical Rainforest Based on ESDM.” Life 15: 323. 10.3390/life15020323. 40003731 PMC11857670

[ece372612-bib-0084] Zabel, I. H. H. , J. Parrish , A. Moore , and G. Lewis . 2014. “Climate of the Western US.” In The Teacher‐Friendly Guide to the Earth Science of the Western US, edited by M. D. Lucas , R. M. Ross , and A. N. Swaby . Paleontological Research Institution.

[ece372612-bib-0085] Zhang, J. , X. Wu , R. Niu , et al. 2012. “Cold‐Resistance Evaluation in 25 Wild Grape Species.” Vitis—Journal of Grapevine Research 51: 153–160.

[ece372612-bib-0086] Zhang, L. , E. Marguerit , L. Rossdeutsch , N. Ollat , and G. A. Gambetta . 2016. “The Influence of Grapevine Rootstocks on Scion Growth and Drought Resistance.” Theoretical and Experimental Plant Physiology 28: 143–157. 10.1007/s40626-016-0070-x.

[ece372612-bib-0087] Zhang, M. , X. Xu , T. Zhang , et al. 2025. “The Dynamics of Wild *Vitis* Species in Response to Climate Change Facilitate the Breeding of Grapevine and Its Rootstocks With Climate Resilience.” Horticulture Research 12: uhaf104. 10.1093/hr/uhaf104. 40406503 PMC12096287

[ece372612-bib-0088] Ziter, C. , E. A. Robinson , and J. A. Newman . 2012. “Climate Change and Voltinism in Californian Insect Pest Species: Sensitivity to Location, Scenario and Climate Model Choice.” Global Change Biology 18: 2771–2780. 10.1111/j.1365-2486.2012.02748.x. 24501055

[ece372612-bib-0089] Zizka, A. , D. Silvestro , T. Andermann , et al. 2019. “CoordinateCleaner: Standardized Cleaning of Occurrence Records From Biological Collection Databases.” Methods in Ecology and Evolution 10: 744–751. 10.1111/2041-210X.13152.

